# Supplementary material for: Group‐level brain decoding with deep learning
Source: Hum Brain Mapp. 2023 Sep 27;44(17):6105–19. doi: 10.1002/hbm.26500 (PMC10619368; doi:10.1002/hbm.26500)
Supplement: Supplementary file 1 — Data S1: Supporting Information. [file HBM-44-6105-s001.pdf]

Supplementary Material

Dropout was set to 0.4 and 0.7, and a batch size of 590 and 59 was used for group-level and subject-level models, respectively. The learning rate was set to 0.0001 for group-level, and 0.00005 for subject-level models. Training of a single subject-level and group-level model took 5-15 minutes and 4 hours on an NVIDIA A100 GPU, respectively. For linear models, validation losses (cross-entropy) and accuracies were negatively correlated, i.e. loss decreases while accuracy increases, and eventually both suggested overfitting. Since non-linear models are more expressive, they overfitted sooner according to the loss, but accuracy kept improving until it reached a plateau, never overfitting. Analysing the loss distribution across validation examples (for non-linear models) shows that even during overfitting most examples' loss keeps decreasing with a few high-loss outliers disproportionately influencing the mean. Since accuracy is binary, outliers are diminished, explaining the apparent difference in learning behaviour. For linear models, this unintuitive behaviour was not observed probably due to inherent model simplicity.

We analysed our main findings on another publicly available visual MEG dataset with 92 different images[1] (15 subjects, and 30 trials per image). Linear subject-level models achieved 35% accuracy, whereas a linear group model without embeddings had 12%, and a nonlinear group model with embeddings had 28%. Thus we can see that our approach behaves similarly on this dataset, improving a lot over the naive group baseline, but not quite achieving the performance of the linear SL models. Finetuning the group model separately on individual subjects achieved 38% accuracy surpassing from-scratch SL models.

To gain further insight into the learned subject embeddings we computed accuracy on each subject's validation data using other subjects' embeddings. In the resulting subject-by-subject confusion matrix the value in the *i*-th row and *j*-th column shows how well the embedding of subject *i* can be replaced with the embedding of subject *j* (Figure 1). After division with the original accuracies, the metric shows how much accuracy can be retained when swapping subject embeddings. Some subjects' embedding cannot be replaced by others (e.g. subject 3), and some subjects' embedding can be more easily replaced (e.g. subject 12). Conversely, some subjects' embeddings are more general as they can replace many others (e.g. subject 14), and some are less general (e.g. subject 2). We tried clustering this matrix, and looked at correlation with both embedding distance and subject accuracy, however no meaningful results were found.

We compared the accuracy-based PFI analysis with a standard gradient-based analysis where

[1] `http://userpage.fu-berlin.de/rmcichy/fusion_project_page/main.html`

	E0	E1	E2	E3	E4	E5	E6	E7	E8	E9	E10	E11
V0		0.16	0.09	0.08	0.26	0.21	0.13	0.12	0.13	0.18	0.12	0.10
V1	0.13		0.04	0.11	0.11	0.09	0.10	0.13	0.11	0.18	0.12	0.10
V2	0.09	0.05		0.14	0.06	0.10	0.08	0.10	0.10	0.11	0.13	0.10
V3	0.04	0.04	0.07		0.03	0.06	0.03	0.05	0.05	0.06	0.06	0.06
V4	0.36	0.16	0.12	0.07		0.17	0.33	0.24	0.13	0.25	0.16	0.16
V5	0.15	0.08	0.08	0.11	0.11		0.09	0.16	0.18	0.14	0.08	0.08
V6	0.14	0.11	0.05	0.05	0.23	0.08		0.09	0.09	0.15	0.08	0.12
V7	0.23	0.16	0.17	0.13	0.26	0.29	0.21		0.24	0.21	0.26	0.33
V8	0.15	0.10	0.05	0.07	0.11	0.11	0.07	0.17		0.13	0.14	0.13
V9	0.25	0.24	0.12	0.12	0.19	0.15	0.19	0.20	0.16		0.27	0.16
V10	0.29	0.13	0.11	0.10	0.16	0.15	0.15	0.24	0.24	0.25		0.15
V11	0.11	0.15	0.05	0.10	0.18	0.12	0.10	0.21	0.13	0.19	0.10	
V12	0.60	0.30	0.23	0.19	0.40	0.49	0.30	0.37	0.47	0.42	0.49	0.42
V13	0.10	0.06	0.05	0.05	0.14	0.07	0.10	0.10	0.07	0.04	0.06	0.14
V14	0.19	0.10	0.05	0.06	0.30	0.10	0.29	0.15	0.10	0.20	0.12	0.17

Figure 1: Subject embedding confusion matrix from the trained `non-linear group-emb` model. Columns (E0-E14) refer to subject embedding indices and rows (V0-V14) refer to subject validation sets. Greener shading (higher values) shows subjects with higher retained accuracy when their embeddings are swapped.

a salience map is obtained by backpropagating to randomly initialised inputs (Figure 2). We smoothed the temporal profile with the same window size as for the PFI analysis. Temporally we can see that the agreement between the two methods is high, with peaks aligning very well (less than 10ms difference). Spatially the two methods do show some differences, but overall gradient analysis still points to the most important information being in the visual cortex.

Kernel FIR analysis shows the power spectra of kernels' outputs when input examples are Gaussian noise (Figure 3). This analysis is answering a different question about the kernels in WaveNet compared to spectral PFI, which asks what frequency content of the input are kernels most sensitive to. In contrast, Kernel FIR analysis asks what are the input-output filtering characteristics of kernels. This provides more insight into how successive layers in Wavenet build up more and more complex filters. The subject embedding was set to a subject

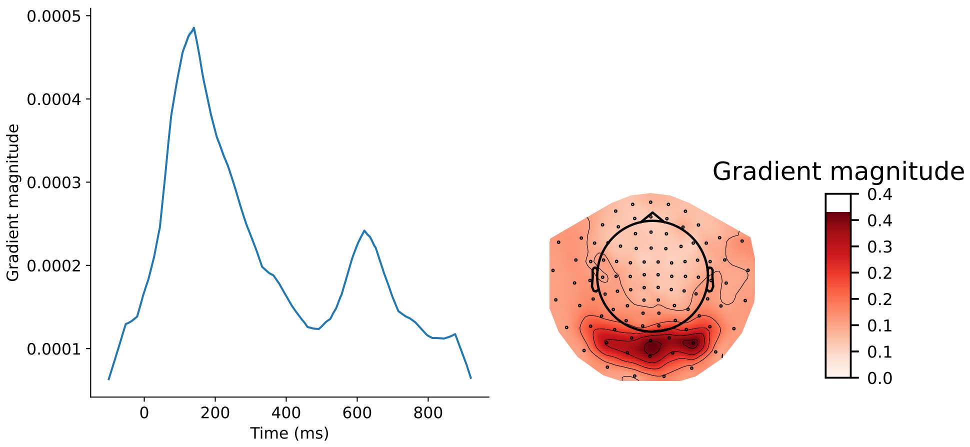

(a) Temporal gradient analysis

(b) Spatial gradient analysis

Figure 2: Using gradient analysis by backpropagating the loss to randomly initialised inputs with the trained `non-linear group-emb` model. In (a) we can see the temporal profile of the gradients averaged over channels. In (b) we can see the spatial profile of the gradients averaged over time.

with average accuracy. The power spectra were normalised to make visual comparisons between kernels easier. Since the WaveNet architecture uses dilated filters with only 2 values per filter, early layers show broad filtering characteristics, but already in layer 2, more emphasis is put on lower frequencies. In deeper layers, filters (kernels) become more tuned to specific frequencies, generally below 20Hz. This is in line with the spectral properties of MEG data as discussed above. Both the spectral PFI and kernel FIR analysis show that there is significant variability between the spectral information encoded by various kernels.

	linear subject	nonlinear subject	nonlinear group-emb
**3 conv layers**	0.45	0.39	0.22
**6 conv layers**	0.41	0.25	0.38

Table 1: Effect of number of convolutional layers on the validation accuracy of two subject-level and one group-level model.

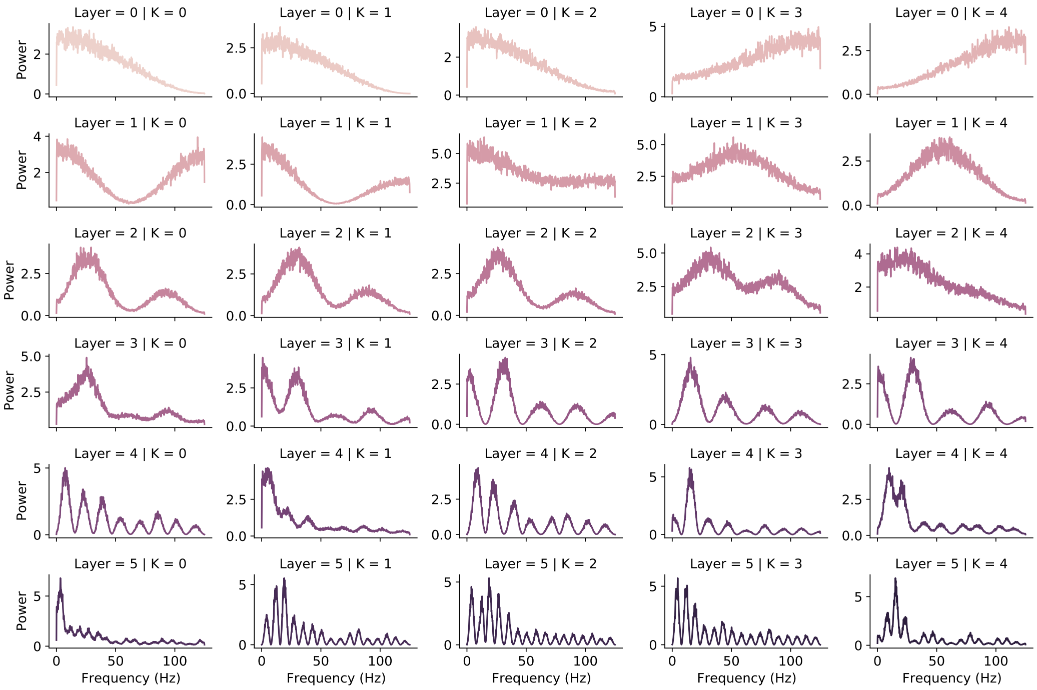

Figure 3: Frequency characteristics of 5 kernels across 6 layers (rows) via kernel FIR analysis in the trained `non-linear group-emb` model. The power spectra are normalised.

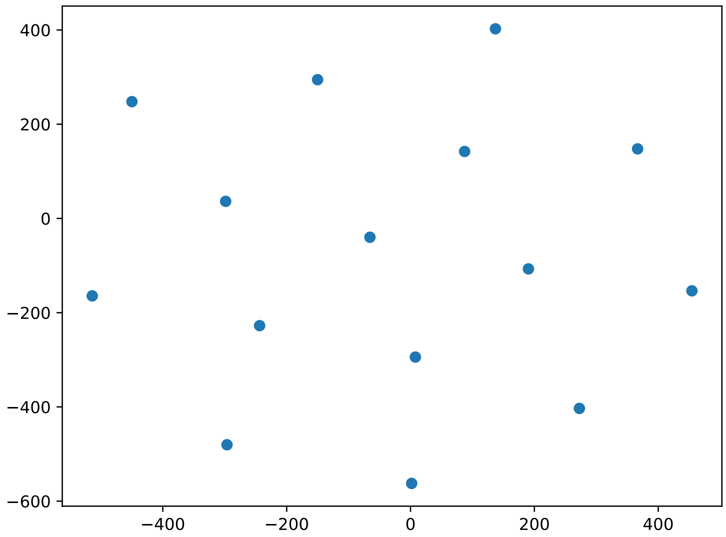

Figure 4: 2D Tsne projection of the subject embeddings in the trained `non-linear group-emb` model.

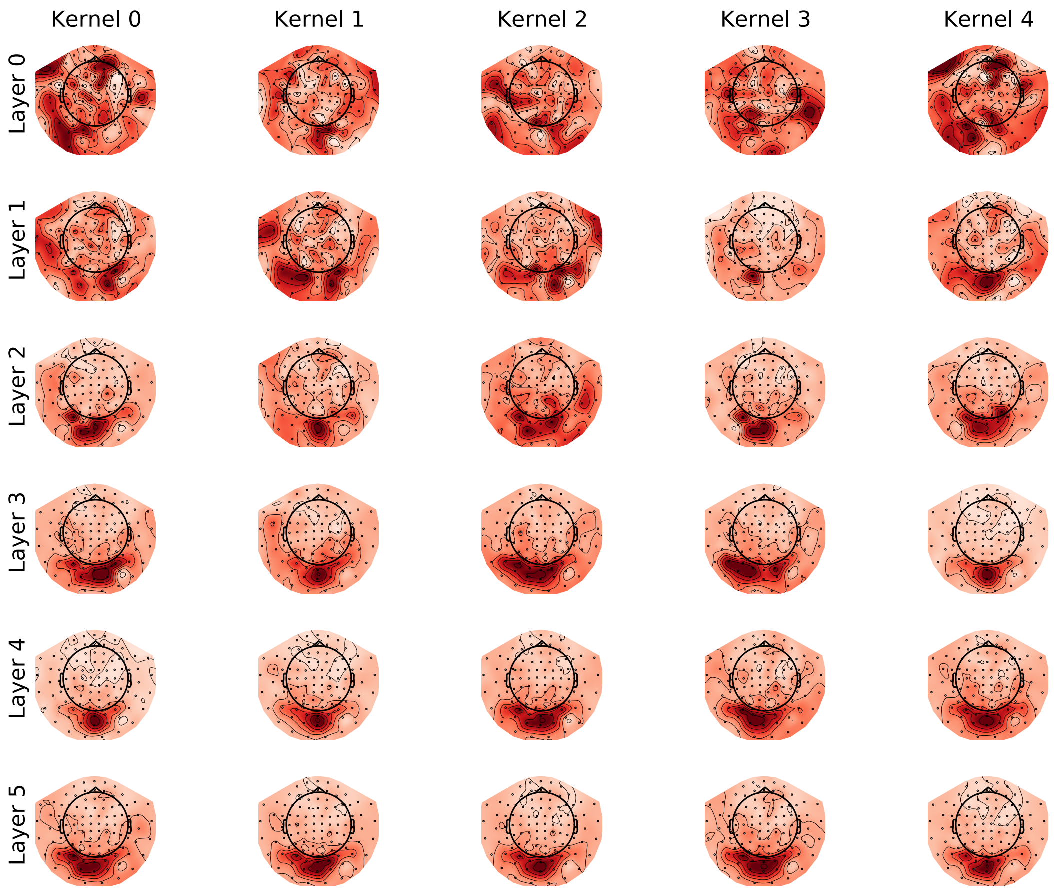

Figure 5: Spatial PFI across 6 layers (rows) in the trained `non-linear group-emb` model, with 5 kernels per row. Darker reds mean higher output deviation.

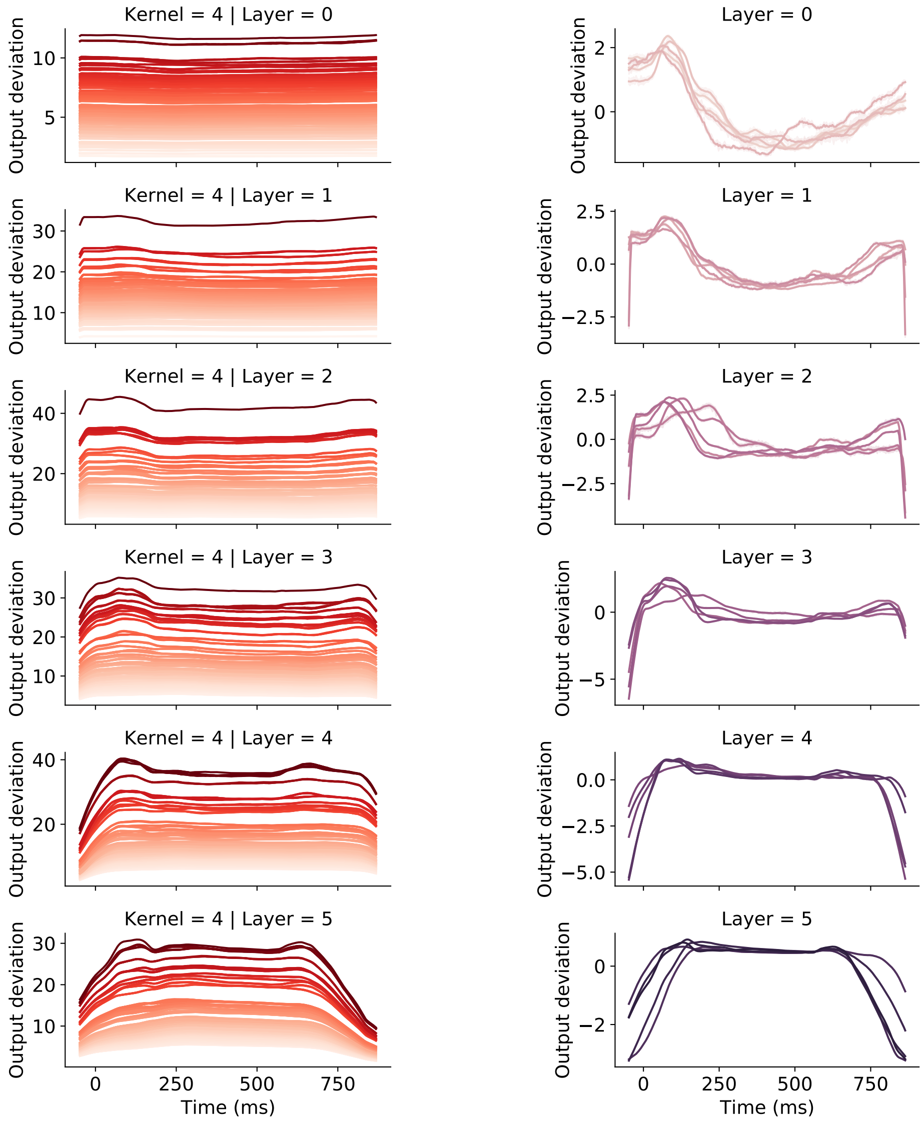

(a) Channel-wise Temporal PFI

(b) Temporal PFI

Figure 6: Channel-wise temporal PFI (a), and temporal PFI (b) across kernels of the `non-linear group-emb` model in 6 layers (rows). For temporal PFI 5 kernels (lines) are plotted together. Channel-wise temporal PFI shows the temporal PFI of each channel for Kernel 5. Channel colouring is matched to the corresponding spatial PFI map, and darker reds mean higher output deviation. For temporal PFI output deviation is normalised. The horizontal axis shows the time elapsed since the image presentation for both temporal PFI types. 95% confidence interval is shown with shading.

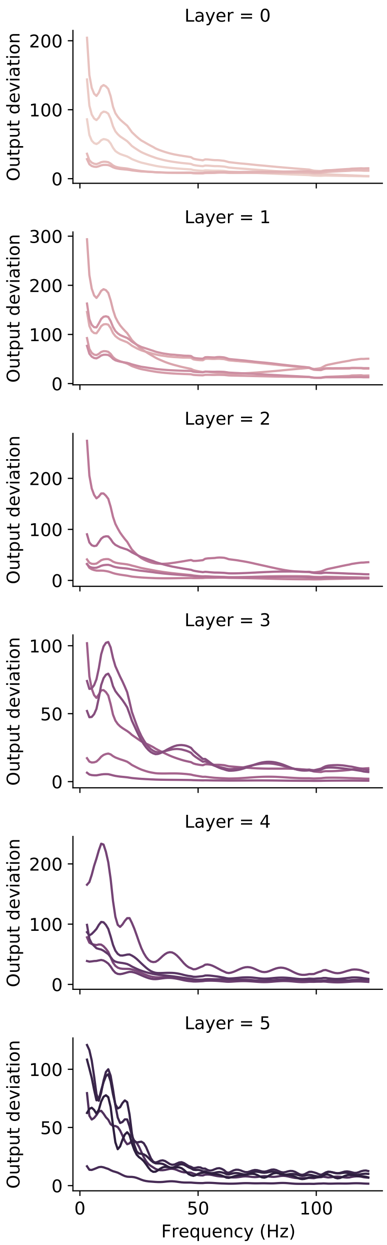

(a) Spectral PFI

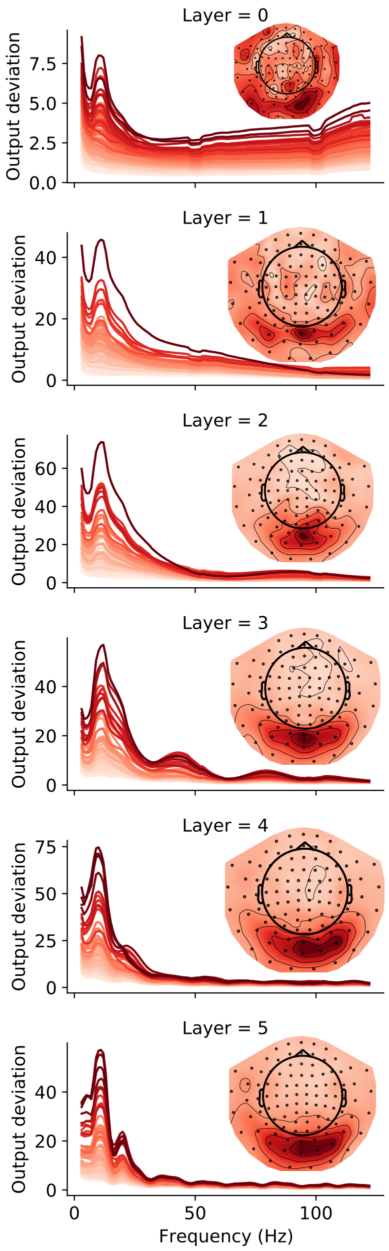

(b) Channel-wise Spectral PFI

Figure 7: Frequency sensitivity of kernels via spectral PFI (a), channel-wise spectral PFI (b) of the trained `non-linear group-emb` model in 6 layers (rows). Kernels are plotted together (lines) for spectral PFI. Each channel-wise spectral PFI plot is for 1 kernel, where lines show the spectral PFI of corresponding channels in the topomap. 95% confidence interval is shown with shading for spectral PFI. Due to small variability across permutations, this is barely visible.
